# The Vacuolar Pathway in Macrophages Plays a Major Role in Antigen Cross-Presentation Induced by the Pore-Forming Protein Sticholysin II Encapsulated Into Liposomes

**DOI:** 10.3389/fimmu.2018.02473

**Published:** 2018-11-05

**Authors:** Yoelys Cruz-Leal, Daniel Grubaugh, Catarina V. Nogueira, Isbel Lopetegui-González, Anaixis del Valle, Felipe Escalona, Rady J. Laborde, Carlos Alvarez, Luis E. Fernández, Michael N. Starnbach, Darren E. Higgins, María E. Lanio

**Affiliations:** ^1^Laboratory of Toxins and Liposomes, Center for Protein Studies, Faculty of Biology, University of Havana, Havana, Cuba; ^2^Department of Microbiology and Immunobiology of Harvard Medical School, Harvard University, Boston, MA, United States; ^3^Department of Biochemistry, Faculty of Biology, University of Havana, Havana, Cuba; ^4^Immunobiology Direction, Center of Molecular Immunology, Havana, Cuba

**Keywords:** antigen cross-presentation, dendritic cells, liposomes, macrophages, sticholysin II, pore-forming protein, cytotoxic immune response

## Abstract

Cross-presentation is an important mechanism for the differentiation of effector cytotoxic T lymphocytes (CTL) from naïve CD8^+^ T-cells, a key response for the clearance of intracellular pathogens and tumors. The liposomal co-encapsulation of the pore-forming protein sticholysin II (StII) with ovalbumin (OVA) (Lp/OVA/StII) induces a powerful OVA-specific CTL activation and an anti-tumor response *in vivo*. However, the pathway through which the StII contained in this preparation is able to induce antigen cross-presentation and the type of professional antigen presenting cells (APCs) involved have not been elucidated. Here, the ability of mouse bone marrow-derived dendritic cells (BM-DCs) and macrophages (BM-MΦs) stimulated with Lp/OVA/StII to activate SIINFEKL-specific B3Z CD8^+^ T cells was evaluated in the presence of selected inhibitors. BM-MΦs, but not BM-DCs were able to induce SIINFEKL-specific B3Z CD8^+^ T cell activation upon stimulation with Lp/OVA/StII. The cross-presentation of OVA was markedly decreased by the lysosome protease inhibitors, leupeptin and cathepsin general inhibitor, while it was unaffected by the proteasome inhibitor epoxomicin. This process was also significantly reduced by phagocytosis and Golgi apparatus function inhibitors, cytochalasin D and brefeldin A, respectively. These results are consistent with the concept that BM-MΦs internalize these liposomes through a phagocytic mechanism resulting in the cross-presentation of the encapsulated OVA by the vacuolar pathway. The contribution of macrophages to the CTL response induced by Lp/OVA/StII *in vivo* was determined by depleting macrophages with clodronate-containing liposomes. CTL induction was almost completely abrogated in mice depleted of macrophages, demonstrating the relevance of these APCs in the antigen cross-presentation induced by this formulation.

## Introduction

Successful immunotherapy against cancer requires the induction of a robust cytotoxic T lymphocyte (CTL) response in combination with the appropriate immune environment and T cell help (1). The initial priming of CTL requires major histocompatibility (MHC) class I antigen presentation by professional antigen presenting cells (APCs) (2). In contrast to “direct-presentation” of protein fragments synthesized within the cell, presentation of exogenous antigens on MHC class I molecules occurs by an alternative route first described by Bevan (3) and later identified as cross-presentation (4, 5). Depending on where the antigen processing takes place, at least two different antigen cross-presentation pathways have been described (4, 6, 7). In the cytosolic pathway, internalized antigens are transferred from the lumen of endocytic compartments to the cytosol where they are degraded by the proteasome. Degradation products are then transported by the transporter associated with antigen processing (TAP) proteins, either to the endoplasmic reticulum (ER) or the lumen of endosomes or phagosomes, allowing formation of peptide-MHC class I complexes (4, 6, 7). In the vacuolar pathway, internalized antigens are degraded within endocytic compartments by lysosomal proteases and loaded onto MHC class I molecules directly in endosomes and phagosomes (4, 6, 7). Antigenic peptides are loaded onto MHC class I molecules with the help of the peptide-loading complex either in the endoplasmic reticulum (ER) or in the phagosomes. ER-resident proteins, including the peptide-loading complex, are transported to phagosomes by a sec22b-dependent mechanism (8). Several studies suggest that there are two routes for MHC class I trafficking to endosomes could coexist: delivery of newly formed MHC class I molecules from the ER and recycling of surface MHC class I molecules (9).

The antigen-presenting cell types most able to provide co-stimulation are dendritic cells (DCs) and macrophages (MΦs). Initially, DCs were thought to be the only cell type competent to prime naive T cells *in vivo* (10). However, several studies demonstrating the ability of macrophages (MΦs) to activate naïve CD8^+^ T lymphocytes both *in vitro* and *in vivo* have since been published (11–13). Moreover, MΦs can play a major role in the antigen cross-presentation of tumor antigens (11) and particulate antigens, specifically liposomal formulations of different compositions (12, 14, 15).

Recently, Laborde et al. demonstrated the ability of liposomes carrying the pore forming protein (PFP) stycholysin II (StII) from the sea anemone *Stichodactyla heliantus*, to induce a strong antigen-specific CTL response in mice (16). This liposomal formulation composed of dipalmitoyl phosphatidylcholine (DPPC) and cholesterol (Chol) in a 1:1 ratio, and co-encapsulating StII with the model antigen ovalbumin (OVA) (Lp/OVA/StII), was also able to induce protection from OVA-expressing tumor challenge in both preventive and therapeutic models (16). These previous findings show the potential of liposomes containing StII as a platform for vaccination to enhance the antigen-specific CTL response of relevance for the cancer immunotherapy or against intracellular pathogens.

The particulate antigens have to be taken up and cross-presented by APCs to initially activate effector CTL, an event known as cross-priming (3–5, 17). In the case of Lp/OVA/StII, the mechanisms by which CTL response are generated against OVA are not completely understood. Namely, which APCs subtypes are responsible for cross-priming naïve CD8^+^ T lymphocytes *in vivo*, as well as the cross presentation pathway enrolled are not known. The aim of this study was to elucidate the pathway by which OVA, co-encapsulated with StII in DPCC:Chol liposomes, induces OVA cross-presentation and which populations of murine APCs are responsible for CD8^+^ T cell activation *in vitro* and cross-priming of CTL *in vivo*. The results provide evidence that bone marrow-derived macrophages (BM-MΦs), but not bone marrow-derived dendritic cells (BM-DCs), are able to cross-present OVA co-encapsulated with StII into liposomes. The antigen cross-presentation in BM-MΦs seems to occur through the vacuolar pathway. In addition, MΦs are responsible for the priming of CTL responses induced by Lp/OVA/StII *in vivo*.

## Materials and methods

### Proteins, lipids, and chemical reagents

OVA grade V used in immunization protocols in soluble form or encapsulated into liposomes and OVA grade II used to coat ELISA plates were purchased from Sigma-Aldrich (St Louis, MO, United States). DPPC and Chol were purchased from Sigma-Aldrich and egg L α- phosphatidylcholine (ePC) from TRANSFERRA Nanosciences Inc. (BC, Canada). StII (Swiss Protein Data Bank P07845) was purified from the sea anemone *Stichodactyla helianthus* and characterized as previously described by Lanio et al. (18). StII protein concentration was determined using the absorption coefficient and its cytolytic activity monitored by a hemolysis assay (18). The protein was stored at −20°C until use. Carboxyfluorescein diacetate succinimidyl ester (CFSE) was acquired from Invitrogen (Paisley, United Kingdom). The immunodominant OVA_257−264_ peptide (SIINFEKL peptide) used in CTL experiments *in vivo* was synthesized by the Center for Genetic Engineering and Biotechnology, Havana, Cuba and stored in phosphate-buffered saline (PBS: Na_2_HPO_4_ 9.6 mM, NaCl 137 mM, KCl 2.7 mM, KH_2_PO_4_ 1.47 mM, pH 7.4) at −20°C until use. The SIINFEKL peptide used in cross-presentation experiments *in vitro* was purchased from Invitrogen (San Diego, CA, United States).

Brefeldin A (BFA) and cytochalasin D were obtained from Sigma-Aldrich. Clodronate for liposome production, epoxomicin, and inhibitors of cathepsin B (CA-O74Me) and cathepsin S (Z-FL-COCHO) were purchased from Calbiochem (San Diego, CA, United States). Cathepsin general inhibitor (Z-FA-fmk) and leupeptin were purchased from ENZO life Science, Inc. (NY, United States) and Invitrogen, respectively.

### Encapsulation of OVA and stii into liposomes

Liposomes encapsulating OVA with or without StII were obtained using a vesicle dehydration and rehydration procedure developed by Kirby and Gregoriadis (19). Briefly, small unilamellar vesicles (SUV) composed of DPPC and equimolar or smaller quantities of Chol (molar ratios 1:1 and 2:1, respectively), were generated by ultrasonication using a Sonics Vibra Cell ultrasonic processor (Sonics & Materials Inc., Newtown, CT, United States) alternating cycles of 30 s of sonication and rest. SUV (19 μmol total lipid) were mixed with 96.2 μg of OVA and 12 μg StII, frozen at −70°C, and lyophilized in an Edwards freezer dryer (Aaron Equipment Company Bensenville, IL, United States) for 24 h. The rehydration step was performed with a small volume of distilled water (50 μl water/16 μmol of lipids) for 30 min above the phase transition temperature of DPPC (45°C), followed by the addition of 0.5 mL of PBS. Non-encapsulated OVA and StII were removed by centrifugation at 10 000 *g* for 15 min (Centrifuge 5415 R, Eppendorf AG, Hamburg, Germany).

### Encapsulation of clodronate into liposomes

Liposomes encapsulating clodronate (Lp/Clodronate) were obtained by a simple dispersion method. The appropriate amounts of lipids ePC and Chol (12 μmol each lipid/per dose) dissolved in chloroform were mixed and evaporated thoroughly at 50°C. Multilamellar vesicles were prepared by hydration of the dried lipid mixture with 120 μg of clodronate dissolved in MilliQ water. Six cycles of freezing and thawing were carried out to improve the clodronate encapsulation and homogenize the vesicle size. The removal of untrapped clodronate was performed by centrifugation at 10,000 g for 15 min (Centrifuge 5415 R, Eppendorf AG). The vesicles corresponding to each mouse administration dose were resuspended in 200 μL of PBS.

### Mice and immunization protocol

Female C57BL/6 (H-2^b^) mice, 6 to 8 weeks of age, were purchased from the Center for Laboratory Animal Production (CENPALAB; Havana, Cuba) and further kept in the animal facilities of the Center of Molecular Immunology (CIM; Havana, Cuba) under standard animal housing conditions. Immunizations were performed subcutaneously (s.c.) in the inferior right limb by delivering 200 μl of liposomal preparation, containing 50 μg of OVA, 6.25 μg of StII and 19 μmol of total lipids (DPPC and Chol) per dose. Two injections of Lp/OVA/StII or PBS with 12 day interval were given.

### Depletion of macrophages

To deplete MΦs in the draining lymph nodes as well as in the blood system and spleen (20), 200 μL of liposomes carrying 12 μg of clodronate (Lp/Clodronate) were injected by intraperitoneal route (i.p.) every 3 days, starting 6 days before the first immunization. One group received 200 μL of liposomes without clodronate as control. To check depletion of MΦs, cell suspensions from the peritoneal cavity (PerC) were pre-harvested and incubated with an anti-CD16/CD32 mAb (BD Biosciences Pharmingen, San Diego, CA, United States) to block Fcγ II/III receptors before staining with fluorochrome-conjugated antibodies. Cells were stained with the following combination of goat anti-mouse antibodies: FITC–CD19, PE-CD11b, PE-Cy5-F4/80, and PE Cy7-CD11c (BD Biosciences Pharmingen), using standard protocols. Cells were acquired with a Gallios flow cytometer (Beckman Coulter, Miami, FL, United States). The analysis was performed using FlowJo 7.2.2 software (Tree Star Ashland, OR, United States). The total number of MΦs was estimated by total cell number in the PerC counted in a Neubauer chamber.

### Bone marrow derived-macrophage and -dendritic cell cultures

Bone-marrow derived MΦs (BM-MΦs) were prepared as previously described by Celada et al. with some modifications (21). Briefly, BM cells were collected from the femurs and tibiae of mice. Cells were plated on non-tissue culture-treated dishes and incubated at 37°C in Dulbecco's modification of Eagle's medium (DMEM) containing 20% heat-inactivated fetal bovine serum (FBS), 30% macrophage colony-stimulating factor (M-CSF)-conditioned medium, 1% penicillin-streptomycin, 1 mM sodium pyruvate, and 2 mM L-Glutamine (Life Technologies Inc., Grand Island, NY, USA). On day 6, cells were harvested and resuspended in DMEM containing 10% fetal bovine serum (FBS) (Gibco™, Waltham, MA, United States) and 15% M-CSF-conditioned medium.

Bone marrow derived-DCs (BM-DCs) were prepared as described in Lutz et al. (22). Briefly, cells were plated on non-tissue culture-treated dishes and incubated at 37°C in RPMI-1640 (Gibco™) supplemented with 10% heat-inactivated FBS LPS-free, 50 mM 2-mercaptoethanol, 1% penicillin-streptomycin, 25 mM HEPES, and 1% GM-CSF (DC medium). On day 2, cells were supplemented with 5 mL of DC medium. On day 4, media was gently removed and fresh DC media was added to the plate. Cells were harvested and used on day 6.

### Detection of antigen cross-presentation by LacZ T cell hybridoma assay

Antigen cross-presentation of the OVA_257−−264_ peptide was detected using the B3Z CD8^+^ T cell hybridoma cell line that expresses β-galactosidase under the control of the IL-2 promoter (15). Briefly, 1 × 10^5^ BM-DCs or 5 × 10^4^ BM-MΦs per well in 96-well flat bottom plates were cultured in RPMI containing antibiotics and 10% (vol/vol) FBS at 37°C in 5% CO_2_. After 24 h, the medium was removed and cells were incubated with or without chemical inhibitors dissolved in RPMI medium without FBS for 30 min before the addition of the antigen. BM-DCs and BM-MΦs were pulsed, in the presence or absence of inhibitors, with OVA in soluble form (OVA) or encapsulated with or without StII into liposomes containing DPPC and Chol in a 1:1 or 2:1 ratio [Lp/OVA/StII (1:1), Lp/OVA/StII (2:1) or Lp/OVA (1:1), Lp/OVA (2:1), respectively] for 90 min at 37°C in 5% CO_2_. BM-DCs and BM-MΦs incubated with the SIINFEKL peptide alone were used as control. After stimulation, cells were washed and co-cultured with 1 × 10^5^ B3Z CD8^+^ T cells at 37°C, 5% CO_2._ After 24 h of incubation the supernatant was removed and 100 μl of LacZ buffer containing 0.15 mM chlorophenol red-β-D-galactopyranoside (Sigma-Aldrich) in PBS plus 0.13% Nonidet P-40, 9 mM MgCl_2_, and 0.1 mM β-mercaptoethanol, were added to the wells. The plates were incubated for 5 h at 37°C. Absorbance was measured at 570/620 nm using a SpectraMax Plate Reader (Tecan, Crailsheim, Germany). IL-2 secretion was measured in cell culture supernatants by ELISA.

### IL-2 quantification by ELISA

To quantify the IL-2 secreted by B3Z CD8^+^ T cells, 96-well polystyrene flat-bottom high binding microtiter plates (Corning™ Costar™, Fisher Scientific, MA, United States) were coated with 10 μg/ml of monoclonal antibodies specific to IL-2 (BD Biosciences Pharmingen), diluted in 0.05 M sodium carbonate buffer pH 9.6, and incubated overnight at 4°C. After washing four times with PBS with 0.05% Tween 20 (PBS/T), the plates were blocked with 10% (v/v) of FBS in PBS (200 μl/well) for 2 h at 37°C. One hundred microliters of culture supernatants diluted 1:2 with 2% FBS in PBS (buffer samples) were added and plates were incubated overnight at 4°C. Serial dilutions (1:2) of recombinant IL-2 were used as standard curve. After four washes with PBS/T, 100 μl of 5 μg/ml of an IL-2-specific biotin-conjugated antibody (clone JE56-5H4, BioLegend, San Diego, CA, United States) was added. Following 2 h of incubation at 37°C, plates were washed six times. Bound antibodies were detected with streptavidin -HRP (BD Biosciences Pharmingen) diluted 1:1,000 in buffer samples. Plates were washed six times and 100 μl of 3,3′,5,5′-tetramethylbenzidine (TMB) substrate solution (Invitrogen) were added. The reaction was stopped with 50 μl of 1 M H_2_SO_4_ after 5 min of incubation and absorbance was measured at 450 nm using a SpectraMax Plate Reader.

### Antigen-specific CTL assay *in vivo*

Total splenocytes from naive C57BL/6 mice were pulsed or not with 1 μM of SIINFEKL peptide for 60 min at 37°C and 5% CO_2_. Peptide-pulsed target cells were extensively washed to remove free peptide. Subsequently, pulsed (target) and non-pulsed (internal control) cells were simultaneously labeled with the fluorescent dye CFSE at 5 μM (CFSE^bright^) and 0.33 μM (CFSE^dull^), respectively, during 5 min at 37°C. Labeled cells were washed, resuspended in PBS, and mixed at 1:1 ratio. A total of 6 × 10^7^ labeled splenocytes in 200 μl of PBS were injected i.v. into immunized mice [Lp/OVA/StII (1:1)] or control mice (PBS) 7 days after the last immunization. Sixteen h later, mice were sacrificed and inguinal lymph nodes closest to the site of immunization were removed, and the total events corresponding to both fluorescent intensities (CFSE^dull^ and CFSE^bright^) were determined by flow cytometry. The percentage of lysis for each mouse was calculated as: % Lysis = 100–[(Total CFSE^bright^ cells/Total CFSE^dull^ cells) _immunized mice_ × 100 × (Total CFSE^dull^ cells/Total CFSE^bright^ cells) _PBS_].

### Ethics statement

All procedures were performed in compliance with the protocols approved by the Institutional Committee for the Care and Use of Laboratory Animals of the CIM (CICUAL, 0018/2009). Animals were sacrificed by cervical dislocation, minimizing their suffering.

### Statistical analysis

Statistical analysis was performed using the SPSS software version 16.0 (SPSS). The Kolmogorov-Smirnov test was used to verify normal distribution of data and the Levene test to determine the homogeneity of variance. Data with normal distribution and equality of variance were analyzed with one-way variance analysis (ANOVA) simple classification, with Dunnett as *post-hoc* test to assess statistical significance between the means of more than two groups. Data not normally distributed or without equality of variance, even after scale transformation, were analyzed using the Kruskal-Wallis non-parametric test with Dunn as *post-hoc* test. For comparing the means of two independent groups with or without normal distribution, the unpaired test and the Mann-Whitney U test were used, respectively.

## Results

### Stii co-encapsulated with OVA into liposomes induces antigen cross-presentation in BM-MΦs but not in BM-DCs *in vitro*

**Figure 1:**
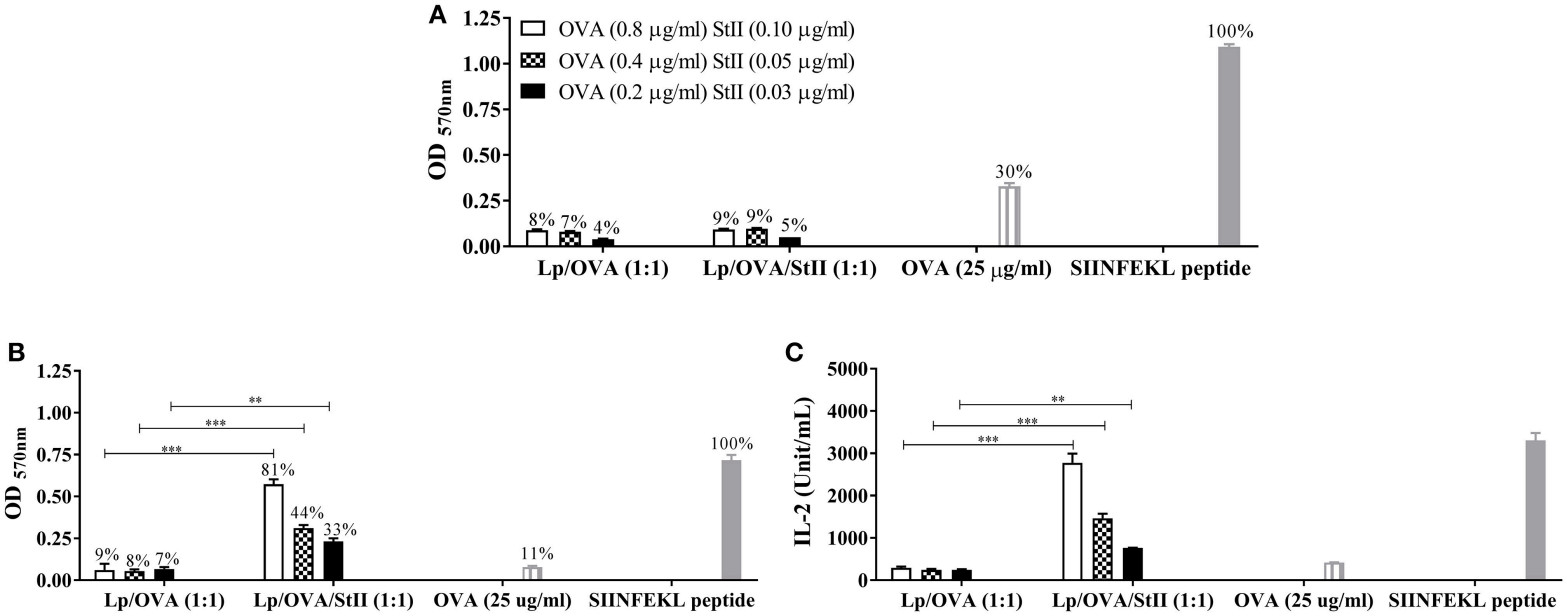
StII co-encapsulated with OVA into liposomes induces the antigen cross-presentation in bone marrow-derived macrophages (BM-MΦs) but not in bone marrow-derived dendritic cells (BM-DCs). **(A)** Bone marrow-derived DCs (BM-DCs) and **(B,C)** Bone marrow-derived macrophages (BM-MΦs) from C57BL/6 mice were incubated with the indicated concentrations of OVA co-encapsulated with or without StII into DPPC:Chol liposomes (1:1) [Lp/OVA/StII (1:1) and Lp/OVA (1:1), respectively]. Both APC incubated with either 25 μg/ml of soluble OVA and 10 nM of SIINFEKL peptide were used as controls. After stimulation, cells were washed and co-cultured with OVA-specific B3Z CD8^+^ T cells. β-galactosidase activity of the B3Z cells was measured by a colorimetric assay. **(A,B)** Mean ± SEM of optical density at 570 nm (OD _570nm_) of four independent experiments performed with BM-DCs and BM-MΦs, respectively, are shown. Efficiency of antigen cross-presentation with respect to the SIINFEKL peptide is also shown above each bar. **(C)** IL-2 concentration in culture supernatants from BM-MΦs was measured by ELISA. Statistical analysis was performed with the Mann–Whitney *U*-test: ^**^p < 0.01, ^***^p < 0.001.

StII encapsulated with the model antigen OVA into liposomes composed by DPPC:Chol in a 1:1 molar ratio induced a strong CD8^+^ T cell proliferation and CTL response *in vivo* (16). To investigate the APCs involved in this response, we evaluated the ability of BM-DCs and BM-MΦs to activate SIINFEKL-specific B3Z CD8^+^ T cell hybridoma line after stimulation with different concentrations of OVA encapsulated with or without StII into liposomes. Unexpectedly, BM-DCs stimulated with Lp/OVA and Lp/OVA/StII were only able to induce low level of CD8^+^ T cell activation reaching only a 4–9% relative at total cells activated by BM-DCs stimulated with SIINFEKL peptide used as a control (Figure 1A). BM-DCs only induced a significant activation of CD8^+^ T cells when they were stimulated with 25 μg/ml of free OVA (30%) (Figure 1A). However, lower quantities of the soluble antigen, similar to those encapsulated into liposomes, did not induce antigen cross-presentation (data not shown). The highest concentration assessed of OVA encapsulated into liposomes with or without StII did not induce noticeable secretion of IL-2 by BM-DCs, while OVA in soluble form and particularly SIINFEKL peptide were able to induce significant levels of this cytokine (Supplementary Figure 1). In contrast, BM-MΦs were clearly able to activate B3Z CD8^+^ T cells when they were stimulated with Lp/OVA/StII (Figure 1B). The CD8^+^ T cell activation levels induced by BM-MΦs pulsed with Lp/OVA/StII were significantly higher than those detected when these cells were stimulated with Lp/OVA (Figure 1B). In addition, BM-MΦs pulsed with Lp/OVA/StII induced a robust secretion of IL-2 by the B3Z CD8^+^ T cells, being this significantly higher than the level induced by these APCs upon Lp/OVA stimulation (Figure 1C). BM-MΦs stimulated with the SIINFEKL also induced a secretion of IL-2 by the B3Z CD8^+^ T cells (Figure 1C). Notably, a much higher concentration of free OVA was required to achieve CD8^+^ T cell activation by BM-DCs. In contrast, BM-MΦs did not induce a significant activation of CD8^+^ T cells even in the presence of this high concentration of free antigen. To figure out why BM-DCs were unable to induce antigen cross-presentation after Lp/OVA/StII stimulation, antigen internalization experiments with BM-DCs and BM-MΦs stimulated with OVA labeled with FITC encapsulated into liposomes with or without StII and in soluble form were performed. BM-DCs were almost unable to internalize OVA encapsulated into liposomes independently of the StII presence and the range of OVA concentrations evaluated (Lp/OVA/StII and Lp/OVA) (Supplementary Figure 2A). In contrast, high percentages of BM-MΦs that internalized the antigen upon stimulation with both liposomes formulations were detected (Supplementary Figure 2B). Both kinds of APCs stimulated with high concentration of OVA in soluble form (25 μg/mL) were able to uptake the antigen.

**Figure 2:**
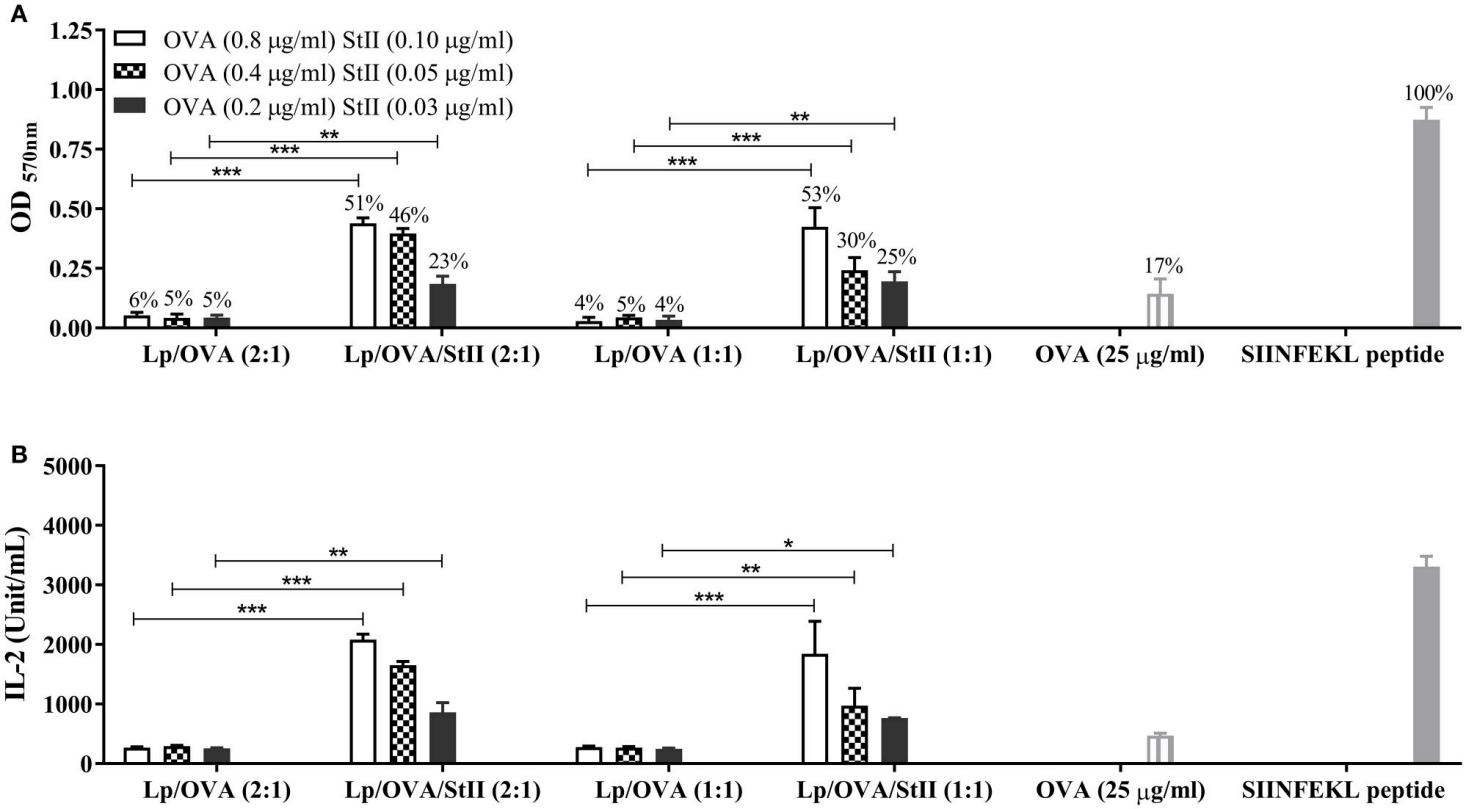
StII co-encapsulated with OVA into liposomes with two different ratios of cholesterol induced antigen cross-presentation in BM-MΦs. Bone marrow-derived macrophages (BM-MΦs) from C57BL/6 mice were cultured for 90 min with the indicated concentration of OVA co-encapsulated or not with StII into liposomes containing different ratios of DPPC and Chol, [Lp/OVA/StII (2:1), Lp/OVA (2:1), Lp/OVA/StII (1:1), Lp/OVA (1:1)]. BM-MΦs stimulated with 25 μg/ml soluble OVA and 10 nM SIINFEKL peptide were used as experimental controls. After stimulation, cells were washed and co-cultured with OVA-specific B3Z CD8^+^ T cells. β-galactosidase activity of the B3Z cells was assessed by a colorimetric assay. **(A)** Mean ± SEM of OD _570nm_ of three independent experiments are shown. Efficiency of antigen cross-presentation with respect to the SIINFEKL peptide is also shown above each bar. **(B)** IL-2 concentration in culture supernatants was measured by ELISA. Means ± SEM of two independent experiments are shown. Statistical analysis was performed with the Mann-Whitney U-test: ^*^p < 0.05, ^**^p < 0.01, ^***^p < 0.001.

Chol has a pronounced effect on the thermotropic phase behavior and organization of DPPC bilayers (23, 24). The high stability of DPPC liposomes containing Chol has been demonstrated and explained by the formation of stronger condensed binary complexes in these lipid layers (25). Previous results obtained in our group demonstrated that co-encapsulated StII with OVA into liposomes composed of DPPC and Chol in a 1:1 ratio (Lp/OVA/StII (1:1) induced higher level of cross-priming of CD8^+^ T lymphocyte *in vivo* than liposomes containing half quantity of Chol (Lp/OVA/StII (2:1) (manuscript in preparation). To explore the influence of reducing the Chol amount in the DPPC liposomes on the ability of StII to induce OVA cross-presentation in BM-MΦs, we compared the CD8^+^ T cell activation by BM-MΦs stimulated with these two liposome formulations. Irrespectively of the level of Chol in the formulations, the presence of StII enhanced the CD8^+^ T cell activation (Figure 2A). In addition, high levels of IL-2 were secreted by CD8^+^ T lymphocytes activated by BM-MΦs stimulated with OVA co-encapsulated with StII in both liposomal formulations (Figure 2B). However, liposomes with the same lipid compositions but without StII failed to induce a significant CD8^+^ T cell activation corroborating the importance of this PFP for this process (Figure 2).

In summary, only BM-MΦ stimulated with liposomal formulations containing StII co-encapsulated with the antigen were able to induce a significant antigen cross-presentation, which indicate the major role of BM-MΦ in this process and the ability of this PFP to promote antigen presenting in the MHC class I context. Given these findings; further experiments to elucidate the pathway through which Lp/OVA/StII induce antigen cross-presentation in BM-MΦs were performed.

### Liposomes co-encapsulating stii with OVA are internalized by BM-MΦ through phagocytosis and/or macropinocytosis

**Figure 3:**
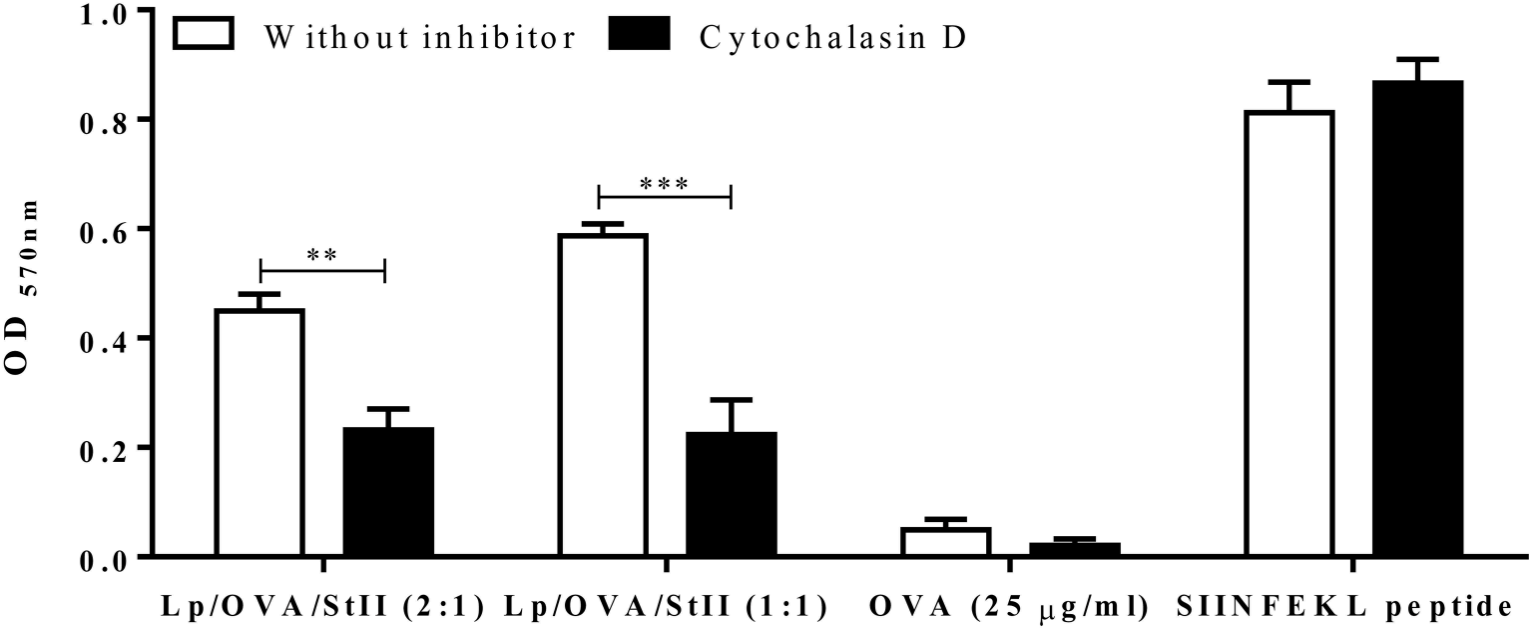
Cytochalasin D inhibits antigen cross-presentation induced by StII co-encapsulated with OVA into liposomes. Bone marrow-derived macrophages (BM-MΦs) from C57BL/6 mice were pre-incubated or not with 10 μM cytochalasin D for 30 min before stimulation. Next, the cells were stimulated with OVA co-encapsulated with StII into liposomes containing DPPC and Chol in 2:1 or 1:1 ratios [Lp/OVA/StII (2:1) or Lp/OVA/StII (1:1)] at final concentrations of 0.8 μg/ml and 0.1 μg/ml of each protein, respectively in the presence or absence of the inhibitor. Cells stimulated with 25 μg/ml OVA in soluble form and 10 nM of SIINFEKL peptide in similar conditions were used as controls. After incubation with the stimuli, cells were washed and co-cultured with B3Z CD8^+^ T cells. β-galactosidase activity of the B3Z cells was assessed by a colorimetric assay. Mean ± SEM of OD _570nm_ of two independent experiments are shown. Statistical analysis was performed with the Unpaired *t*-test: ^**^p < 0.01, ^***^p < 0.001.

The internalization route of exogenous antigen by APCs would determine the antigen processing and presenting pathway (26–28). To examine whether Lp/OVA/StII were internalized by a mechanism that involve actin polymerization such as: phagocytosis and macropinocytosis; BM-MΦs were stimulated with Lp/OVA/StII (2:1) or Lp/OVA/StII (1:1) in the presence of cytochalasin D. Soluble OVA and the SIINFEKL peptide were used as controls. The ability of the stimulated BM-MΦs to activate the CD8^+^ T cells was significantly reduced by cytochalasin D treatment (Figure 3). This clearly establishes the phagocytosis and/or macropinocytosis as the one of the routes in BM-MΦs for internalizing antigens encapsulated into these liposomes.

### Lysosome proteases inhibitors but not the proteasome inhibitor block cross-presentation of OVA co-encapsulated with stii into liposomes

**Figure 4:**
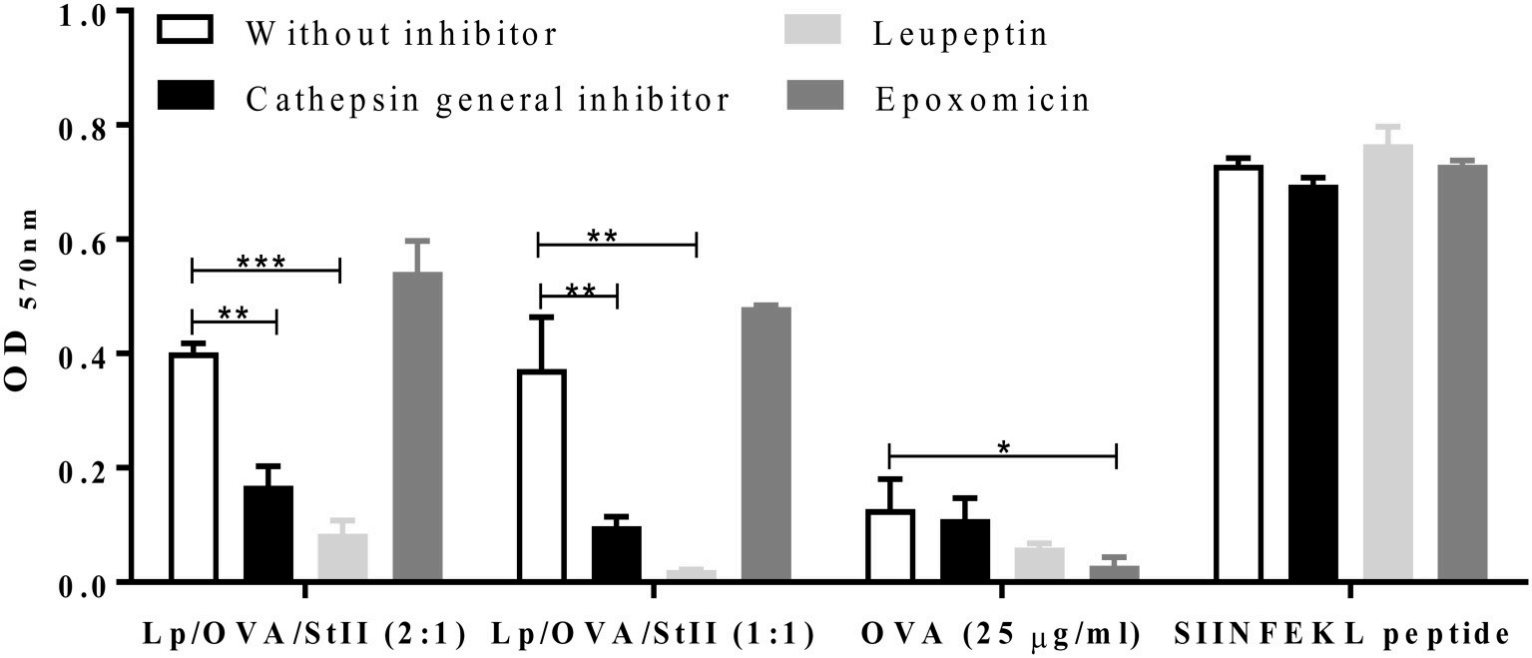
Lysosome proteases but not proteasome inhibitors decrease antigen cross-presentation induced by StII co-encapsulated with OVA into liposomes. Bone marrow-derived macrophages (BM-MΦs) from C57BL/6 mice were pre-incubated or not with 50 μM of lysosome protease inhibitors, leupeptin, and a cathepsin general inhibitor or with 1 μM of the proteasome inhibitor epoxomicin and stimulated with OVA co-encapsulated with StII into liposomes composed DPPC and Chol in 2:1 or 1:1 ratios [Lp/OVA/StII (2:1) or Lp/OVA/StII (1:1)] at final concentrations of 0.8 and 0.1 μg/ml of each protein, respectively. Cells stimulated with 25 μg/ml of OVA in soluble form and 10 nM of the SIINFEKL peptide were used as controls. Cells were washed and co-cultured with B3Z CD8^+^ T cells. β-galactosidase activity of the B3Z cells was determined by a colorimetric assay. Mean and SEM of OD _570nm_ of two independent experiments are shown. Statistical analysis was performed with the one-way ANOVA test with Dunnett post-test: ^*^p < 0.05, ^**^p < 0.01, ^***^p < 0.001.

In the classical MHC class I pathway, cytosolic proteins are cleaved by the proteasome before MHC class I loading. Cross-presentation of exogenous antigens on MHC class I can also involve transport into the cytosol followed by proteasomal processing or degradation by lysosomal proteases in the endosomal compartment (4, 6, 10). To determine the pathway by which OVA co-encapsulated with StII in DPPC:Chol liposomes is cross-presente, antigen cross-presentation assays in the presence of proteasome and lysosome proteases inhibitors were performed. BM-MΦs were first treated with the proteasome inhibitor epoxomicin before pulsing with two types of liposomes: Lp/OVA/StII (2:1) and Lp/OVA/StII (1:1). Unexpectedly, the treatment with epoxomicin had no effect on antigen cross-presentation from either Lp/OVA/StII formulation (Figure 4). A similar behavior was observed with liposomes carrying a lower concentration of both proteins (Supplementary Figure 3). In contrast, epoxomicin inhibited the cross-presentation of soluble OVA, demonstrating that proteasome inhibition was effective under our experimental conditions (Figure 4).

To elucidate if Lp/OVA/StII antigen processing takes place in the endosomal compartment, BM-MΦs were pre-incubated with a cathepsin general inhibitor or leupeptin, a reversible inhibitor of cysteine, serine, and threonine proteases. Treatment with the cathepsin general inhibitor or leupeptin markedly decreased the cross-presentation of OVA co-encapsulated with StII in both liposomal compositions (Figure 4). Cathepsin general inhibitor reduced antigen cross-presentation of Lp/OVA/StII (2:1) and Lp/OVA/StII (1:1) by 60 and 74%, respectively. Also, leupeptin induced a strong inhibition in both cases (80 and 94%, respectively). Similar effects of the cathepsin general inhibitor or leupeptin were observed when BM-MΦs were stimulated with liposomes encapsulating lower concentrations of both proteins (Supplementary Figure 3). MHC class I presentation on of OVA in soluble form at 25 μg/ml and exogenous SIINFELK peptide by BM-MΦs were unaffected by these inhibitors (Figure 4).

Overall, these data indicate that encapsulation of OVA with StII into theses liposomes leads to lysosomal but not cytosolic degradation, which demonstrates that StII carried by these liposomal formulations induces antigen cross-presentation by the vacuolar pathway.

### Cathepsin proteases differentially affect cross-presentation of OVA co-encapsulated with stii into liposomes with different chol amounts

**Figure 5:**
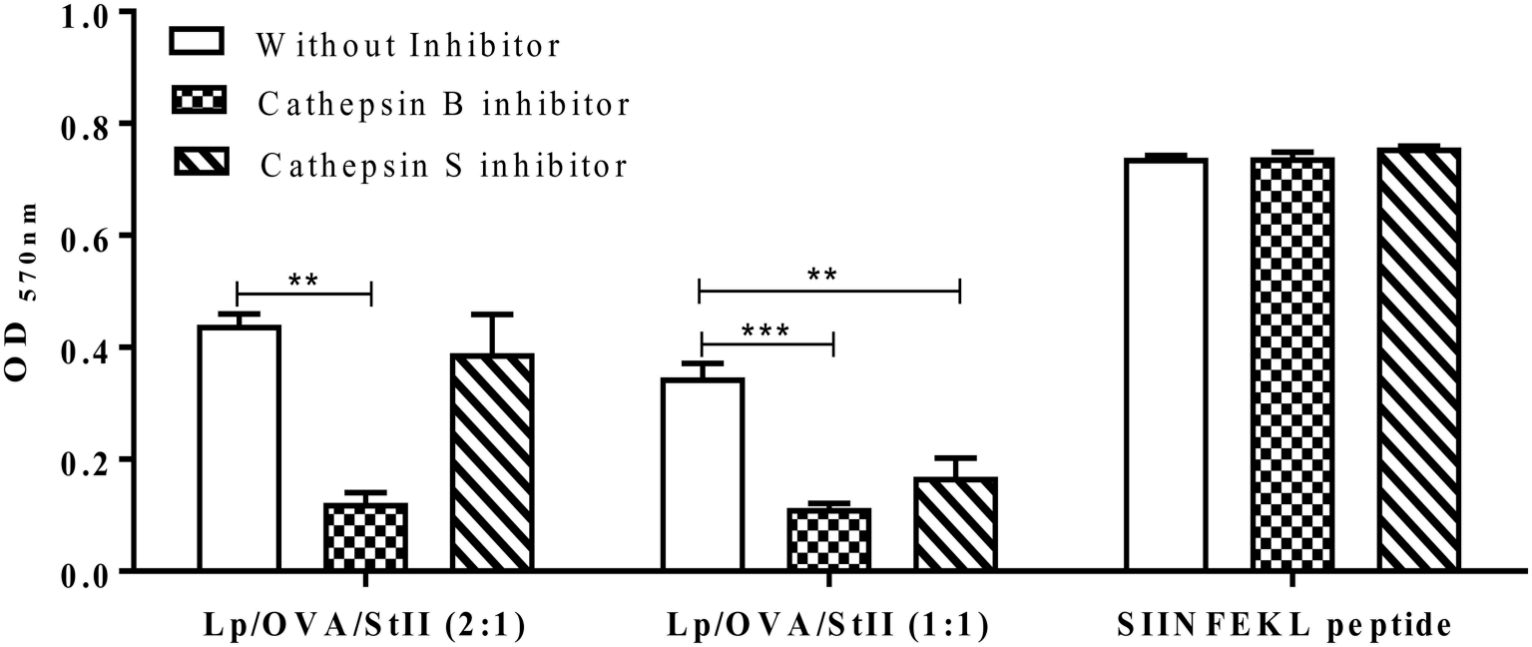
Cathepsin B and S differentially affect antigen cross-presentation induced by StII co-encapsulated with OVA into liposomes containing different quantities of Chol. Bone marrow-derived macrophages (BM-MΦs) from C57BL/6 mice were pre-incubated or not with 10 μM cathepsin B or cathepsin S inhibitors and stimulated with OVA co-encapsulated with StII into liposomes composed by DPPC and Chol in 2:1 or 1:1 ratios [Lp/OVA/StII (2:1) or Lp/OVA/StII (1:1)] at final concentrations of 0.8 μg/ml and 0.1 μg/ml of each protein, respectively. Cells stimulated with 10 nM of the SIINFEKL peptide were used as experimental controls. After stimulation cells were washed and co-cultured with B3Z CD8^+^ T cells. β-galactosidase activity of the B3Z cells was measured by a colorimetric assay. Mean ± SEM of OD _570nm_ of two independent experiments are shown. Statistical analysis was performed with one-way ANOVA test with Dunnett post-test: ^**^p < 0.01, ^***^p < 0.001.

The cathepsin general inhibitor affected the cross-presentation of OVA co-encapsulated with StII into both liposomal formulations. To determine which cathepsins were involved in the processing of OVA for MHC class I presentation, inhibitors specific for the cysteinyl proteases cathepsin S and cathepsin B were tested. MHC class I presentation of Lp/OVA/StII (1:1) was blocked by both cathepsin S and B inhibitors. However, the cross-presentation induced by Lp/OVA/StII (2:1) was only affected by the cathepsin B inhibitor (Figure 5). These results could be suggesting that the proteolytic processing of antigen carried by these two liposomal formulations occurs at different maturation stages of phagosomes; however future experiments to explain this finding should be performed. As expected, presentation of the SIINFEKL peptide was not affected by any of these inhibitors (Figure 5).

### Golgi function is involved in antigen cross-presentation by stii-containing liposomes

**Figure 6:**
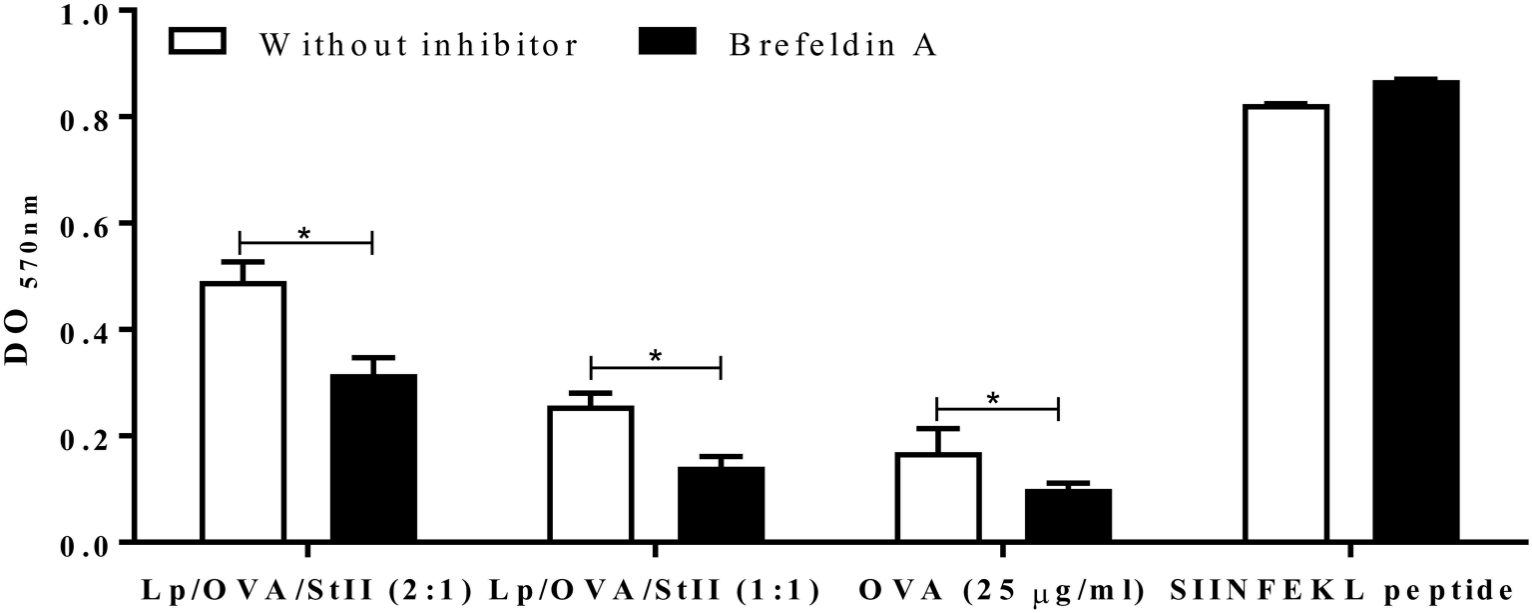
Nascent MHC class I molecules are partially required for antigen cross-presentation induced by StII co-encapsulated with OVA into liposomes. Bone marrow-derived macrophages (BM-MΦs) were pre-incubated with 5 μg/mL brefeldin A (BFA) and stimulated with OVA co-encapsulated with StII into liposomes composed by DPPC and Chol in 2:1 or 1:1 ratios [Lp/OVA/StII (2:1) and Lp/OVA/StII (1:1)] at final concentrations of 0.8 μg/ml and 0.1 μg/ml of each protein, respectively. After stimulation cells were washed and co-cultured with B3Z CD8^+^ T cells. β-galactosidase activity of the B3Z CD8^+^ T cells was determined by a colorimetric assay. Mean and SEM of OD _570nm_ of two independent experiments are shown. Statistical analysis was performed with Mann–Whitney U-test: ^*^p < 0.05.

Processing and presentation of both endogenous and exogenous antigens often requires nascent MHC molecules (29), but can be also used MHC molecules recycling from the cell surface (30). To determine if presentation of OVA co-encapsulated into liposomes with StII involves newly synthesized molecules of MHC class I, brefeldin A (BFA) was used to block protein transport from the Golgi apparatus. BFA treatment significantly blocked MHC class I antigen presentation of Lp/OVA/StII (2:1) and Lp/OVA/StII (1:1) (36 and 50%, respectively) (Figure 6). However, this inhibition was incomplete, suggesting that the newly synthesized MHC class I molecules are not the exclusive source for antigen cross-presentation induced by liposomes containing OVA with StII. As control, the effect of BFA on cross-presentation of soluble OVA was tested. As in previous reports (31), in BFA-treated APCs the presentation of soluble OVA on MHC class I was inhibited (Figure 6).

### Macrophages are essential for cross-presenting OVA co-encapsulated with stii into liposomes *in vivo*

**Figure 7:**
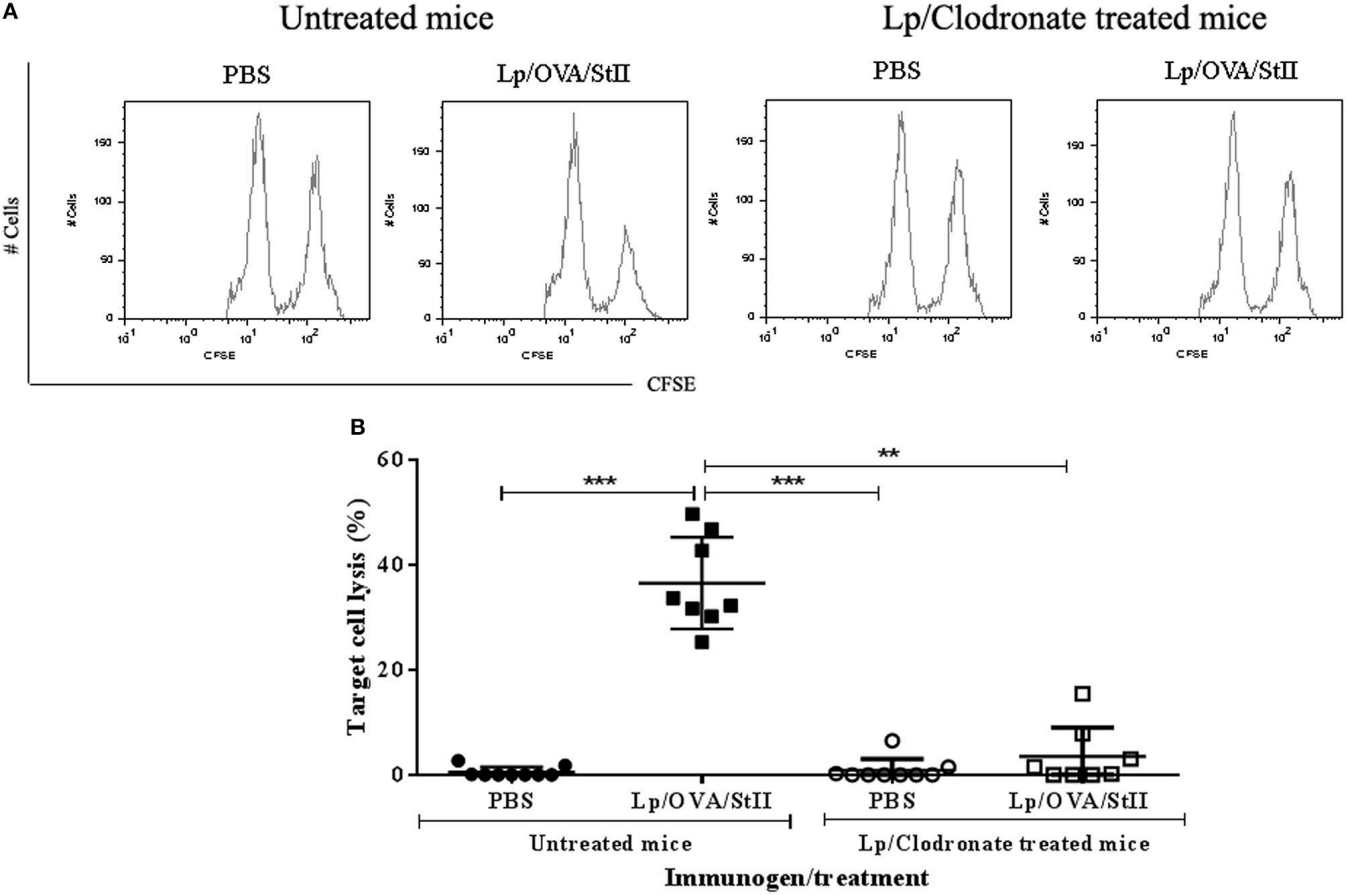
Macrophage depletion abrogates *in vivo* cytotoxic T lymphocytes response induced by liposomes co-encapsulating OVA with StII. C57BL/6 mice were depleted of macrophages by i.p. injection of liposomes containing clodronate. Mice treated or not with clodronate were immunized s.c. twice at days 0 and 12 with 50 μg of OVA co-encapsulated with 6.25 μg StII into DPPC:Chol liposomes (1:1). PBS was administered as control. Seven days after the second immunization, mice received i.v. 6 × 10^7^ cells containing an 1:1 proportion of target cells (SIINFEKL-charged and CFSE^bright^ -labeled splenocytes) and control cells (without peptide and CFSE^dull^-labeled splenocytes). Sixteen hours after injection of the cells, mice were sacrificed and inguinal draining lymph nodes were harvested. Total events corresponding to both fluorescent intensities (CFSE^dull^ and CFSE^bright^) were determined by FACS. The percentage of specific lysis was calculated as: 100–[(Total CFSE^bright^ cells/Total CFSE^dull^ cells) _immunized mice_ × 100 × (Total CFSE^dull^ cells/Total CFSE^bright^ cells) _PBS_]. **(A)** Histograms of CFSE^dull^ and CFSE^bright^ cells from an individual mouse representative of each experimental group. **(B)** Percentages of target cell lysis in individual animals from multiple CTL assays *in vivo*. Statistical analysis was performed with the Kruskal-Wallis test with Dunn post-test ^**^p < 0.01, ^***^p < 0.001.

The co-encapsulation of StII with OVA in DPPC:Chol (1:1) liposomes leads to CD8^+^ T lymphocyte proliferation and CTL differentiation *in vivo* (16). After showing the general capability of BM-MΦs to cross-present OVA co-encapsulated with StII into liposomes *in vitro*, the role of macrophages in the CTL induced by this liposomal formulation *in vivo* was studied. C57BL/6 mice were depleted of macrophages by administration of liposomes containing clodronate. Peritoneal cell analysis by flow cytometry showed that approximately 85% of total macrophages were depleted. Contour graph representative of macrophages population analysis from Lp/Clodronate treated and untreated mice were showed in Supplementary Figure 4. A histogram representative of one mouse from each experimental group of the *in vivo* cytotoxicity assay is shown in Figure 7A, where a reduction of the CFSE^bright^ cells pulsed with the SIINKEKL peptide compared to those CFSE^dull^ without peptide can be observed, in mice immunized with Lp/OVA/StII (1:1). In contrast, mice depleted of macrophages and immunized with Lp/OVA/StII (1:1) exhibited similar quantities of both cell populations (Figure 7A). A quantitative analysis of the percentage of lysed target cells indicated that macrophage depletion provoked a significant reduction of OVA-specific CTL (~90%) (Figure 7B), which demonstrates the relevance of this APCs for cross-priming of CD8^+^ T lymphocytes *in vivo*.

## Discussion

The co-encapsulation StII with a model antigen into liposomes has been shown to induce an antigen-specific CD8^+^ T cell response and potent anti-tumor immunity resulting in a promising vaccine platform for cancer immunotherapy (16). However, the mechanism through which this PFP induces antigen cross-presentation and which APCs are mainly involved in this process remained unknown. In this report, we show that DPPC:Chol liposomes co-encapsulating OVA and the PFP StII (Lp/OVA/StII) are efficiently internalized by phagocytosis and/or macropinocytosis and presented on MHC class I molecules by BM-MΦs but not by BM-DCs *in vitro*. We were able to confirm that macrophages play a primary role in cross-priming CTL induced by Lp/OVA/StII *in vivo*. The results also show that MHC class I peptide generation from Lp/OVA/StII is mediated by lysosomal proteases and not by the proteasome, indicating the preferential endosomal localization of these particles. Nascent MHC class I molecules seem to be also involved in the antigen cross-presentation induced by Lp/OVA/StII.

The fact that BM-MΦs are able to cross-present peptides from Lp/OVA/StII (Figures 1B,C) is in agreement with other *in vitro* studies which have shown a requirement of macrophages in cross-presentation of several particulate antigens (12, 32–34). Particularly, our results demonstrate that BM-DCs stimulated with Lp/OVA/StII were unable to activate the SIINFEKL-specific B3Z CD8^+^ T cell hybridoma line (Figure 1A and Supplementary Figure 1). This deficiency could be due to the inability of these APCs to internalize or process peptides from Lp/OVA/StII. The lower phagocytic activity of BM-DCs in comparison with BM-MΦs has been well established (30). BM-DCs exhibit better internalization capacity of antigens in soluble form or of nanometric sizes (35). The results obtained demonstrated that BM-DCs exhibited a lower ability to internalize OVA antigen upon Lp/OVA and Lp/OVA/StII stimulation, unlike of BM-MΦs that were able to internalize OVA more efficiently after stimulation with both liposomes formulations (Supplementary Figure 2). The average size of the liposomes obtained by the procedure described here corresponds to those previously reported (36, 37) and ranges from 1.6 to 2.2 μm (16). Then, the large size of these vesicles could have limited their internalization by BM-DCs. However, BM-DCs stimulated with Lp/OVA/StII were completely unable to activate the B3Z CD8^+^ T cells; suggesting that inefficient internalization cannot be the only cause of the inability of DCs to process and present antigen from Lp/OVA/StII. In addition, the ability of DCs to cross-present antigens from micrometric size particles has been reported. In contrast to Lp/OVA/StII, most of these formulations promote antigen delivery into the cytosol where processing is mediated by the proteasome (31, 38). In other cases where antigens are linked or absorbed on the surface of particles, they are more accessible to lysosomal proteases and their degradation preferentially occurs in phagosome compartments (31). In the formulations assessed here, there is only a small fraction of the antigen exposed at the vesicle surface (16). Thus, the degradation of encapsulated OVA would require disassembly of the incorporated DPPC:Chol liposomes, a process more complex than the degradation of an antigen bound to the surface.

On the other hand, antigen degradation in the endocytic pathway is dependent on proteolytic enzymes whose activation is pH-dependent (38, 39). Even though BM-DCs maintain an alkaline pH in their endocytic compartment through the production of reactive oxygen species (ROS), thereby restricting antigen degradation and potentially allowing more molecules to be available for cytosol exportation and cross-presentation by this pathway (40), this could be not convenient for particles as Lp/OVA/StII. Macrophages have been shown to rapidly acidify their phagosomes, thus leading to the destruction of internalized antigens (40, 41). In addition, there is a marked difference in lysosomal protease composition between DCs and MΦs *in vivo* or *in vitro* differentiated from bone marrow precursors (41). Also, there are differences between these APCs populations regarding the content of cathepsins (42); which we showed that are important for the degradation of the OVA carried in this liposomal formulation (Figures 4, 5). For instance, cathepsin S is significantly more expressed in BM-MΦs and peritoneal MΦs than in BM-DCs and splenic DCs, while cathepsin B has only been detected in MΦs (41). Since we found these proteases to play a major role in the generation of the OVA peptide from Lp/OVA/StII (Figure 5), their lower levels of expression in BM-DCs could also explain these cells' inability to cross-present this antigen. Further work would be also performed to completely elucidate why BM-DCs are unable to efficiently cross present Lp/OVA/StII.

Cross-presentation of antigen from Lp/OVA/StII was selectively inhibited by cytochalasin D (Figure 3), suggesting that this process requires the internalization of these particles by phagocytosis and/or macropinocytosis. However, the incomplete inhibition of the antigen cross-presentation observed in BM-MΦs stimulated with liposomes upon cytochalasin D treatment suggest that other mechanisms of antigen uptake can be involved. These observations are consistent with the phagocytic antigen processing pathway for presenting bacterial antigens in MHC class I molecules (43). A similar pathway may be followed by several formulations based on particulate antigens that not penetrate actively into the cytosol, including acid-resistant liposomes (33, 34), mammalian cells (44) and OVA conjugated to beads (32). Even though this pathway is more efficient for particulate antigens, high doses of soluble antigen can also induce MHC class I presentation (28, 45, 46). OVA co-encapsulated with StII into liposomes is cross-presented more efficiently when compared to the soluble antigen. This increased capacity to be presented by MHC class I molecules might be due to enhanced antigen uptake showed by macrophages (Supplementary Figure 2). Alternatively, the release of antigens from the phagosomes to the MHC class I pathway might be more efficient than from other endocytic compartments. In agreement with this, Houde et al. (47) and Ackerman et al. (17) demonstrated that phagosomes display the elements and properties needed to be self-sufficient for the cross-presentation of exogenous antigens. The findings obtained here and those previously published by other authors (27), suggest that favoring the entry of exogenous antigens through phagocytosis could improve the efficacy of active immunotherapy strategies against cancer.

OVA cross-presentation induced by Lp/OVA/StII was unaffected by the proteasome inhibitor epoxomicin but was prominently suppressed by inhibitors of lysosomal proteases (cathepsin general inhibitor and leupeptin) (Figure 4). These results demonstrate that OVA co-encapsulated with StII into liposomes was processed by proteasome-independent and lysosome-dependent manners, consistent with the proposed cross-presentation vacuolar pathway. These findings also suggest that StII does not promote delivery of the antigen to cytosol at least when it is carried out by these kinds of liposomes. Surprisingly, the mechanism through which Lp/OVA/StII improves the antigen cross-presentation and CTL generation *in vivo* is different from the mechanism reported for other PFP/antigen/liposome formulations. Specifically, in the case of listeriolysin O (LLO), the hemolytic protein of *Listeria monocytogenes*, carried with the OVA into liposomes the antigen cross-presentation is mediated by the cytosolic pathway (48–51). Our results are consistent with those obtained by Mant et al. (31), who showed that cross-presentation from small particles but not for larger ones is sensitive to a proteasome inhibitor, indicating that the pathway of cross-presentation is also influenced by the particle size of phagocytosed antigen. The specific internalization route may dictate the use of the vacuolar pathway. Indeed, the cross-presentation of encapsulated, but not bead-associated polymer microspheres or viral antigens was shown to be cathepsin S-dependent (42). Furthermore, TLR2 agonist-dipalmitoylated peptides are internalized TLR2 via clathrin-mediated endocytosis and their presentation by MHC class I molecules was blocked in the presence of a lysosomal degradation inhibitor; but the unconjugated form of the peptides was cross-presented through the cytosolic pathway (26).

While the antigen cross-presentation induced by both liposomal/StII formulations was sensitive to lysosomal protease inhibitors (Figure 4), these events were differentially affected by particular cathepsin S and B inhibitors (Figure 5). During the phagolysosome biogenesis, the nascent phagosome matures by fission and partial fusion with endocytic compartments in “*kiss and run*” events, acquiring superior degradative properties. Formation and subsequent maturation of phagosomes in APCs are related to the characteristics of the ingested particulate antigens. Degradation of the phagocytosed particulate vaccines generates peptide antigens for loading on MHC molecules and subsequent antigen presentation at the cell surface (38). Since both cathepsin inhibitors decreased the antigen cross-presentation induced by Lp/OVA/StII (1:1), the processing of antigen carried by this liposomal formulation might be occurring along the maturation pathway of phagosomes interacting with different endocytic compartments. In contrast, the antigen cross-presentation induced by Lp/OVA/StII (2:1) was only affected by the cathepsin B inhibitor, which would indicate a preferential processing of antigen in specific phagosomes, maybe in early stage of their maturation, where the hydrolytic capacity is lesser (38, 52). The phagosome maturation seems to be characterized by the sequential acquisition of hydrolases. Cathepsin S is barely detectable in early phagosomes but its level continues to increase up to 24 h, while most of the hydrolases seem to reach their highest level at 6 h after phagosome formation (53, 54). The results obtained in this work could be probably linked to the differential stability of DPPC liposomes when the Chol ratio varies (25). Interestingly, both liposomal formulations were able to enhance in a similar degree the antigen cross-presentation *in vitro* (Figure 2), however Lp/OVA/StII (2:1) exhibited a decreased ability to induce CTL *in vivo* (manuscript in preparation).

The results obtained here show the partial inhibition of the cross-presentation of Lp/OVA/StII by BFA (Figure 6), suggesting that new MHC class I molecules seems to be involved in this process. However, the fact that inhibition by BFA was partial prompts that recycling MHC class I molecules perhaps play an important role in the presentation of this antigen. Similarly, a partial inhibition (35%) of the presentation of complexes generated after phagocytosis of OVA-latex beads has been described (47). Also, the reported sensitivity of antigen cross-presentation to BFA inhibition is variable, ranging from a complete blockage in BM-MΦs (55, 56) and DC 2.4 dendritic-like cells (57) to only partial inhibition in BMA3.1A MΦs (47), a minor inhibition with prolonged exposure to high concentrations in peritoneal MΦs (58); or no inhibition in peritoneal MΦs (59, 60). Such studies employed a wide size range of particulate antigens, from 0.5 to 3 μm (47, 56, 59). Becker et al. (61) found that the ER membranes were selectively recruited to phagosomes when J774 MΦs phagocyte large (3 μm) but not small (0.8 μm) particles. Newly formed MHC class I molecules can be transported to phagosomes through interaction with CD74 (62), and surface MHC class I molecules can accumulate into Rab11a-containing recycling endosomes, which are recruited to phagosomes upon Toll-like receptor (TLR) signaling (63). Interestingly, StII has the ability to maturate DCs (16) in a TLR4/MyD88-depending manner, which contributes to the CTL response generated by StII-containing liposomes (manuscript in preparation). Beside phagocytic receptors, co-receptors such as TLRs also seem to influence the phagosomal maturation in favor of antigen presentation and induction of an immune response (63, 64). Similarly MΦs activation by StII could occur and promote the recruitment of surface MHC class I molecules in phagosomes. Further studies should be performed to demonstrate this hypothesis.

The MΦ role in antigen cross-presentation induced by Lp/OVA/StII *in vitro* was also assessed *in vivo* through MΦs depletion with liposomes encapsulating clodronate. The lower percentage of CTL induced by Lp/OVA/StII in mice depleted of MΦs (Figure 7) suggested the main role of these APCs in this process. A previous study also described the role of these cells in the cross-presentation of particulate antigens (12). We cannot completely discard the role of other phagocytic cells in the antigen cross-presentation induced by Lp/OVA/StII, since the depleting macrophages with clodronate-containing liposomes induce that all professional phagocytes are depleted, including dendritic cells (65). However, the inability of DCs to cross-present Lp/OVA/StII *in vitro* suggests a decrease relevance of these cells in the *in vivo* process. Nevertheless, future experiments with specific exhaustion of DCs should be carried out in order to determine their contribution at the CTL *in vivo* response induced by Lp/OVA/StII.

In conclusion, our study provides evidence that OVA co-encapsulated with StII into liposomes is internalized by phagocytosis and cross-presented through the vacuolar pathway by BM-MΦs but not by BM-DCs. Moreover, in the CTL priming induced by the liposomal formulation containing StII *in vivo*, macrophages played a key role. These events described in this work should be determining the efficacy of the liposomal formulation encapsulating StII as a vaccine platform for enhancing CTL response. Nevertheless, further studies are required to completely elucidate the mechanism underlying the enhancement of antigen cross-presentation induced by StII carried by liposomes.

## Author contributions

YC-L Conception and design of the work, acquisition, analysis and interpretation of the data and writing of the manuscript. DG Acquisition, analysis and interpretation of the data and critical revision of the manuscript. CN Acquisition, analysis and interpretation of the data and critical revision of the manuscript. IL-G Acquisition, analysis, interpretation of data and revision of the manuscript. AdV Acquisition, analysis and interpretation of data. FE Acquisition, analysis and interpretation of data. RL Analysis, interpretation of data and revision the manuscript. CA Analysis and interpretation of the data and revision of the manuscript. LF Critical scientific support, interpretation of the data, revision of the manuscript. MS Critical scientific support, interpretation of the data. DH Critical scientific support, interpretation of the data, revision of the manuscript and final approval of the manuscript. ML Conception and design of the work, interpretation of the data, writing, critical revision and final approval of the manuscript.

### Conflict of interest statement

The authors declare that the research was conducted in the absence of any commercial or financial relationships that could be construed as a potential conflict of interest.

## Abbreviations

APCs: Antigen presenting cells
BM-DCs: Bone marrow-derived dendritic cells
BM-MΦs: Bone marrow-derived macrophages
BFA: Brefeldin A
Chol: Cholesterol
CFSE: Carboxyfluorescein diacetate succinimidyl ester
CTL: Cytotoxic T lymphocytes
CW-PSC: Cell Wall Polysaccharide
DMEM: Dulbecco's modification of Eagle's medium
DPPC: Dipalmitoyl phosphatidylcholine
ELISA: Enzyme-linked immunosorbent assay
ePC: Egg L α- phosphatidylcholine
ER: Endoplasmic reticulum
FBS: Fetal bovine serum
FITC: Fluorescein isothiocyanate
FITC-OVA: Ovalbumin labeled with FITC
i. p: Intraperitoneal
Lp/OVA/StII: DPPC and Chol-containing liposomes co-encapsulating OVA with StII
Lp/Clodronate: Liposomes encapsulating clodronate
M-CSF: Macrophage colony-stimulating factor
MΦs: Macrophages
OVA: Ovalbumin
PB: Phosphate buffer
PerC: Peritoneal cavity
PE: Phycoerythrin
PE Cy5.5: Cyanine dye (Cy5.5) combined with phycoerythrin
PE Cy7: Cyanine dye (Cy7) combined with phycoerythrin
SEM: Standard error of the mean
StII: Sticholysin II
s.c: Subcutaneously
SUV: Small unilamellar vesicles
TAP: Transporter associated with antigen processing.
