# Supplementary material for: The Vacuolar Pathway in Macrophages Plays a Major Role in Antigen Cross-Presentation Induced by the Pore-Forming Protein Sticholysin II Encapsulated Into Liposomes
Source: Front Immunol. 2018 Nov 5;9:2473. doi: 10.3389/fimmu.2018.02473 (PMC6230584; doi:10.3389/fimmu.2018.02473)
Supplement: Supplementary file 1 [file Data_Sheet_1.docx]

Supplementary Material

The vacuolar pathway in macrophages plays a major role in antigen cross-presentation induced by the pore-forming protein Sticholysin II encapsulated into liposomes

Yoelys Cruz-Leal^1†^, Daniel Grubaugh^2^, Catarina V. Nogueira^2^, Isbel Lopetegui-González^3^, Anaixis del Valle^3^, Felipe Escalona^1^, Rady J. Laborde^3^, C. Alvarez^1^, Luis E. Fernández^4^, Michael N. Starnbach^2^, Darren E. Higgins^2*^ and María E. Lanio^1*^

Correspondence: Corresponding author: Dr. María Eliana Lanio, [mlanio@fbio.uh.cu](mailto:mlanio@fbio.uh.cu) and Dr. Darren E. Higgins [darren_higgins@hms.harvard.edu](mailto:darren_higgins@hms.harvard.edu)

**Supplementary Materials and Methods**

**Antigen uptake assay by BM-DCs and BM-MΦs**

BM-DCs or BM-MΦs (1 x10^5^ cells/well) were separately seeded in 96-well flat bottom plates for 24 h. Cells were stimulated in the absence of FCS with OVA labeled with FITC and encapsulated with or without StII into liposomes (Lp/FITC-OVA/StII or Lp/FITC-OVA), at different antigen concentrations (from 0.2 to 3.2 μg/mL) for 2 hrs. Free OVA at high concentration (25 μg/mL) and untreated cells as positive and negative control, respectively, were also used. The stimuli were replaced by medium with FCS and the cells were kept for additional 30 minutes. Then the cells were harvested and labeled with anti-CD11c/PerCPCy5.5 or anti-F480/PE mAbs and then fixed with 2% paraformaldehyde. Subsequently, the percentages of FITC+ BM-DCs and BM-MΦs were measured using a Gallios flow cytometer from Beckman Coulter (Indianapolis, USA). Parallel experiments were carried out at 37 ^o^C and 4 ^o^C in order to discard the particles adsorption at cell surface. The percentage of cells that internalize the antigen was calculated as: (% of FITC^+^ cells at 37 ^o^C- % of FITC^+^ cells at 4 ^o^C).

## Supplementary Figures

**Supplementary figure 1.** **StII co-encapsulated with OVA into liposomes did not induce robust secretion of IL-2 by bone marrow-derived dendritic cells (BM-DCs).** BM-DCs from C57BL/6 mice were incubated with 0.8 µg/ml of OVA co-encapsulated with or without StII (0.10 µg/ml) into DPPC:Chol liposomes (1:1) (Lp/OVA/StII (1:1) and Lp/OVA (1:1), respectively). BM-DCs incubated with either 25 µg/ml of soluble OVA and 10 nM of SIINFEKL peptide were used as controls. After stimulation, cells were washed and co-cultured with OVA-specific B3Z CD8^+^ T cells. IL-2 concentration in culture supernatants from BM-DCs was measured by ELISA. Statistical analysis between Lp/OVA and Lp/OVA/StII was performed with the Mann–Whitney U-test.


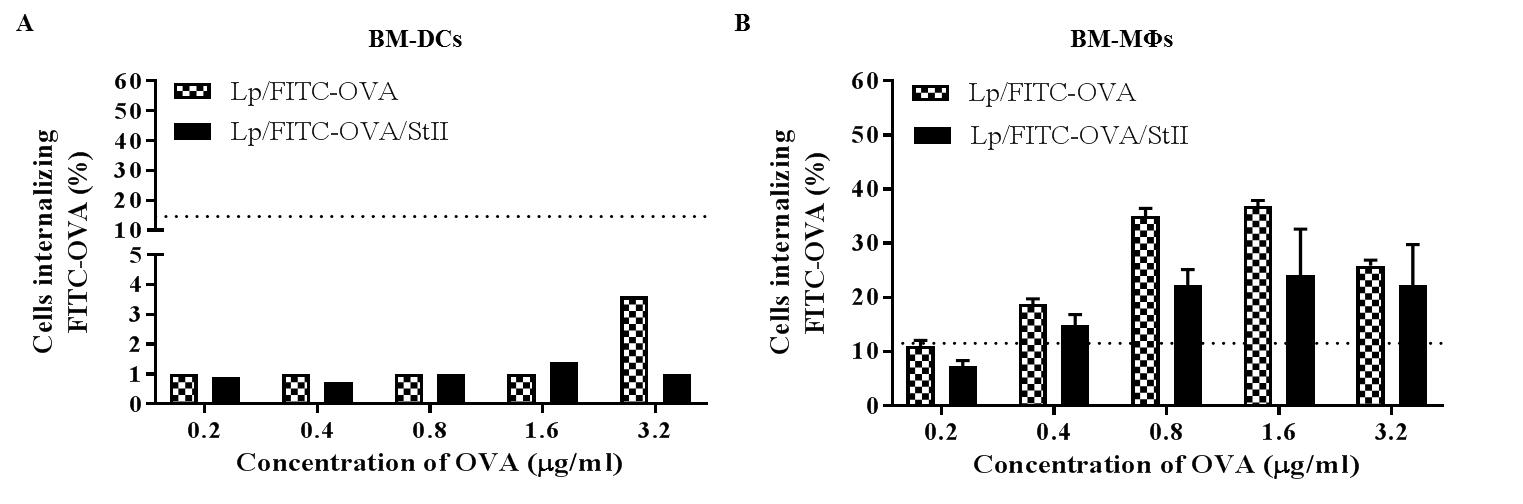


**Supplementary figure 2. BM-MΦs but not BM-DC internalize OVA encapsulated into liposomes with or without StII**. BM-DCs or BM-MΦs (1 x10^5^ cells/well) were stimulated in the absence of FCS with OVA labeled with FITC and encapsulated with or without StII into liposomes (Lp/FITC-OVA/StII or Lp/FITC-OVA), at different antigen concentrations (from 0.2 to 3.2 μg/mL) for 2 hrs. Free FITC-OVA at high concentration (25 μg/mL, dash line) as positive control is also showed. The stimuli were replaced by medium with FCS and the cells were kept for additional 30 minutes. The percentage of BM-DCs (**A**) and BM-MΦs (**B**) that internalized FITC-OVA after Lp/FITC-OVA or Lp/FITC-OVA/StII stimulation were measured by flow cytometry.

**Supplementary figure 3.** **Lysosome proteases but not proteasome inhibitors decrease antigen cross-presentation induced by Lp/OVA/StII encapsulating low quantities of both proteins.** Bone marrow-derived macrophages (BM-MΦs) from C57BL/6 mice were pre-incubated or not with 50 μM of lysosome protease inhibitors, leupeptin, and a cathepsin general inhibitor or with 1 μM of the proteasome inhibitor epoxomicin, followed by stimulation with OVA co-encapsulated with StII into liposomes composed DPPC and Chol in 2:1 or 1:1 ratios [Lp/OVA/StII (2:1) or Lp/OVA/StII (1:1)] at final concentrations of 0.4 µg/ml and 0.05 µg/ml of each protein, respectively. Cells stimulated with 25 µg/ml of OVA in soluble form and 10 nM of the SIINFEKL peptide were used as controls. Cells were washed and co-cultured with B3Z CD8^+^ T cells. β-galactosidase activity of the B3Z cells was determined by a colorimetric assay. Mean and SEM of OD _570nm_ of two independent experiments are shown. Statistical analysis was performed with the one-way ANOVA test with Dunnett post-test: **p*< 0.05, ** *p*< 0.01, *** *p*< 0.001.


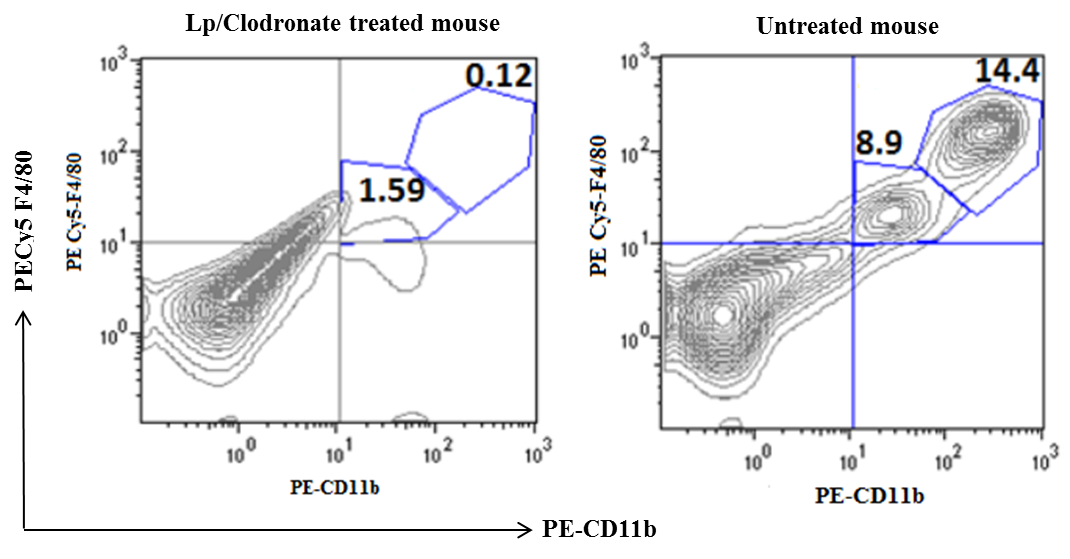


**Supplementary figure 4.** **Lp/clodronate treatment induce successfully macrophage depletion.** C57BL/6 mice were injected by intraperitoneal (i.p.) route with 200 µL of liposomes carrying 12 µg of clodronate (Lp/Clodronate), every 3 days starting six days before the first immunization (Lp/Clodronate treated mouse). Other group of mice received 200 µL of liposomes without clodronate as control (Untreated mouse). To check depletion of MΦs, cell suspensions from the peritoneal cavity were pre-harvested and incubated with the following combination of goat anti-mouse antibodies: FITC–CD19, PE-CD11b and PE-Cy5-F4/80 (BD Biosciences Pharmingen), using standard protocols. Cells were analysis by flow cytometer. After exclusion of CD19^+^ cells, the percentage of both peritoneal macrophages populations were determined (Small peritoneal macrophages (CD11b^low^F4/80^low^) and large peritoneal macrophages (CD11b^high^F4/80^high^).
