# Supplementary figures and images for: The Vacuolar Pathway in Macrophages Plays a Major Role in Antigen Cross-Presentation Induced by the Pore-Forming Protein Sticholysin II Encapsulated Into Liposomes
Source: Front Immunol. 2018 Nov 5;9:2473. doi: 10.3389/fimmu.2018.02473 (PMC6230584; doi:10.3389/fimmu.2018.02473)

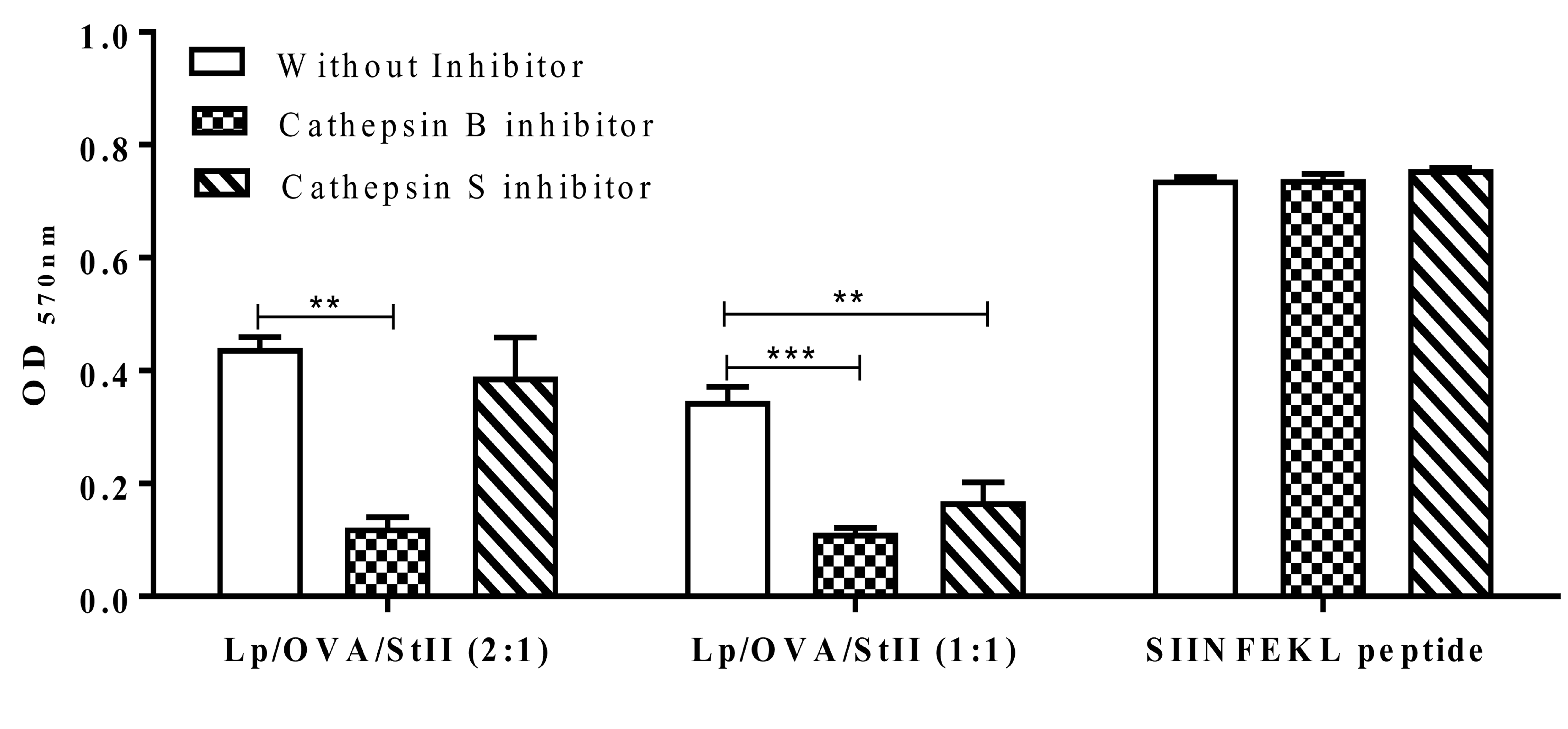

Supplement: Supplementary file 2 [file Figure_5.TIF]

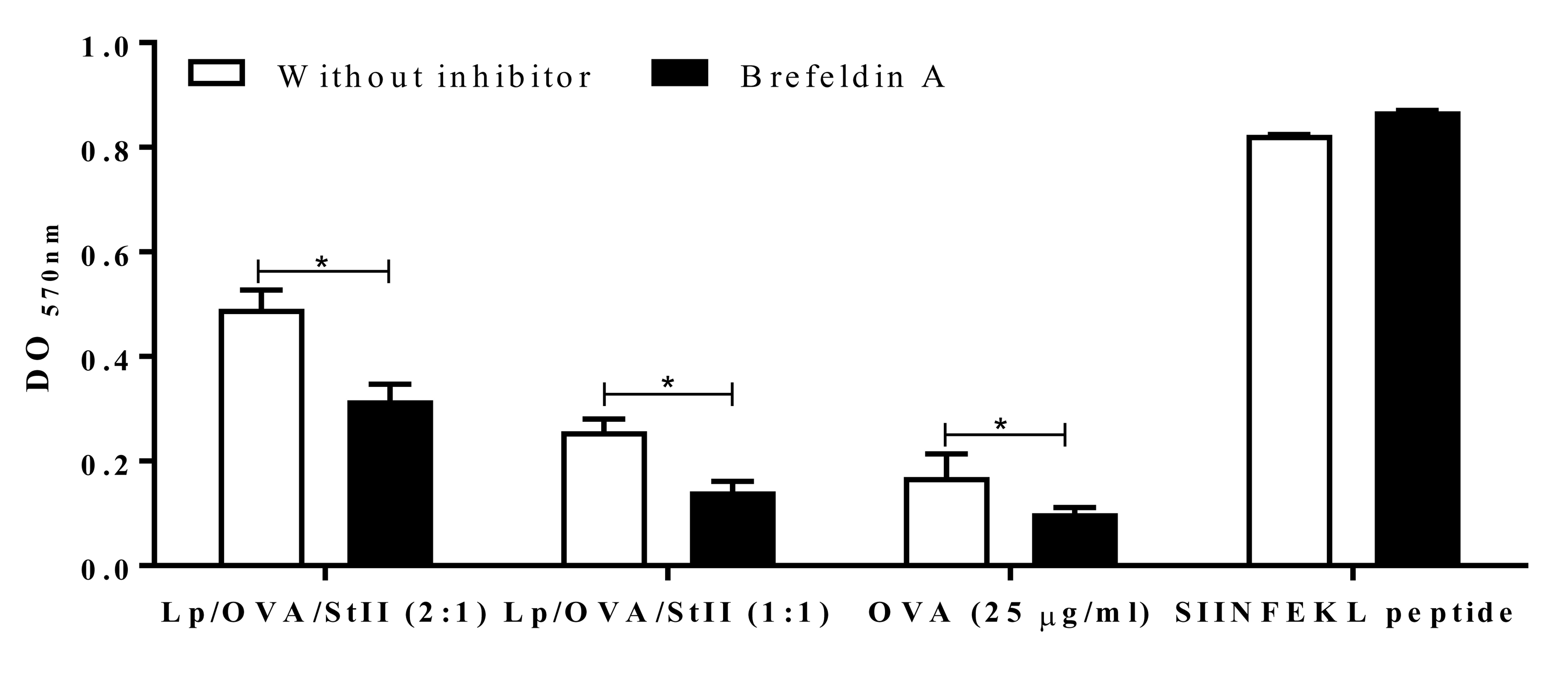

Supplement: Supplementary file 3 [file Figure_6.TIF]
